# A Systematic Review of Circulatory microRNAs in Major Depressive Disorder: Potential Biomarkers for Disease Prognosis

**DOI:** 10.3390/ijms23031294

**Published:** 2022-01-24

**Authors:** Madiha Rasheed, Rabia Asghar, Sundas Firdoos, Nadeem Ahmad, Amina Nazir, Kakar Mohib Ullah, Noumin Li, Fengyuan Zhuang, Zixuan Chen, Yulin Deng

**Affiliations:** 1 Beijing Key Laboratory for Separation and Analysis in Biomedicine and Pharmaceuticals, School of Life Sciences, Beijing Institute of Technology, Beijing 100081, China; madiharasheed@bit.edu.cn (M.R.); rabiaasghar@bit.edu.cn (R.A.); 3820170075@bit.edu.cn (S.F.); mohibullah@luawms.edu.pk (K.M.U.); 6120190133@bit.edu.cn (N.L.); 2 Department of Pharmacy, Abbottabad Campus, COMSATS University Islamabad, Abbottabad 22060, Pakistan; nadeemph@cuiatd.edu.pk; 3 Institute of Animal Science and Veterinary Medicine, Shandong Academy of Agricultural Sciences, Jinan Industry North Road 202, Jinan 250100, China; aminanazir@bit.edu.cn; 4 School of Biology and Medical Engineering, Beihang University, Beijing 100191, China; zhuangfy@buaa.edu.cn

**Keywords:** cimiRNAs, depression, biomarkers, blood, plasma, serum, CSF, neuropsychiatry, perils

## Abstract

Major depressive disorder (MDD) is a neuropsychiatric disorder, which remains challenging to diagnose and manage due to its complex endophenotype. In this aspect, circulatory microRNAs (cimiRNAs) offer great potential as biomarkers and may provide new insights for MDD diagnosis. Therefore, we systemically reviewed the literature to explore various cimiRNAs contributing to MDD diagnosis and underlying molecular pathways. A comprehensive literature survey was conducted, employing four databases from 2012 to January 2021. Out of 1004 records, 157 reports were accessed for eligibility criteria, and 32 reports meeting our inclusion criteria were considered for in-silico analysis. This study identified 99 dysregulated cimiRNAs in MDD patients, out of which 20 cimiRNAs found in multiple reports were selected for in-silico analysis. KEGG pathway analysis indicated activation of ALS, MAPK, p53, and P13K-Akt signaling pathways, while gene ontology analysis demonstrated that most protein targets were associated with transcription. In addition, chromosomal location analysis showed clustering of dysregulated cimiRNAs at proximity 3p22-p21, 9q22.32, and 17q11.2, proposing their coregulation with specific transcription factors primarily involved in MDD physiology. Further analysis of transcription factor sites revealed the existence of HIF-1, REST, and TAL1 in most cimiRNAs. These transcription factors are proposed to target genes linked with MDD, hypothesizing that first-wave cimiRNA dysregulation may trigger the second wave of transcription-wide changes, altering the protein expressions of MDD-affected cells. Overall, this systematic review presented a list of dysregulated cimiRNAs in MDD, notably miR-24-3p, let 7a-5p, miR-26a-5p, miR135a, miR-425-3p, miR-132, miR-124 and miR-16-5p as the most prominent cimiRNAs. However, various constraints did not permit us to make firm conclusions on the clinical significance of these cimiRNAs, suggesting the need for more research on single blood compartment to identify the biomarker potential of consistently dysregulated cimiRNAs in MDD, as well as the therapeutic implications of these in-silico insights.

## 1. Introduction

Major depressive disorder (MDD) is the most prevalent, chronic, and complicated neuropsychiatric disorder of the current era [1,2], causing 800,000 people to commit suicides annually (WHO, 2020). It is characterized by negative sequelae such as low mood, lack of interest and pleasure, fluctuations in weight or appetite, sleep disturbances (insomnia or hypersomnia), fatigue, psychomotor retardation or agitation, remorse, impaired concentration, and lower self-confidence, all of which can lead to suicidal thoughts or attempts [3]. Recently, the World Health Organization (WHO) listed depression as the second leading cause of disability (after cancer) that has burdened nations economically and jeopardized life by affecting over 264 million people worldwide, and plausibly predicted that it would be ranked first by 2030 (WHO, 2020) [4]. Hence, this alarming situation has turned MDD into a major public health concern in developed states.

Despite tremendous efforts, the dilemma underlying the pathophysiology of MDD is still unknown. As a result, approximately 40% of MDD patients do not respond to antidepressant medication and eventually become treatment-resistant as the disease load worsens [2]. Additionally, the lack of specific diagnostic tools (biomarkers) has made MDD more complex when compared with other etiologically related disorders such as bipolar disorder [5]. Most MDD patients are assessed based on clinical symptoms [6] rather than any objective laboratory-based testing, which leads to misdiagnosis or underdiagnosis in primary clinical settings [7]. These clinical diagnostic scales are subjective and may be influenced by intermittent variation in symptoms and apply symptomatic therapies [8,9,10]. Although neuroimaging techniques such as functional magnetic resonance imaging (fMRI) visually assess disease progression, they are still restricted by practicality and high costs [11]. Moreover, various molecular biomarkers such as cytokine [12,13] and GABA levels [8,14] in blood reported diversified outcomes and do not allude to disease progression.

To date, MDD patients are reported to exhibit significant changes in different brain regions compared to healthy subjects. Various studies have reported that synaptic connections and neural, functional, and structural plasticity are impaired, and that brain connectivity is disturbed in MDD subjects [15]. It is proposed that MDD occurs due to systematic changes in the biochemical and signaling pathways regulating moods, cognition, and disposition. Additionally, the complex etiology of MDD is linked with various genetic factors or epigenetic factors that have become leading causes of disrupted neurological mechanisms, including neurogenesis, neuronal apoptosis, synaptic plasticity, etc. [16]. Although this domain has been extensively investigated in modern research, a deep understanding of impaired or compromised cellular pathways involved in MDD is required to fine-tune current strategies for MDD patients. However, converging evidence suggests that no single mechanism can fully explain and include the etiopathogenesis of MDD [17]. Moreover, heterogeneity in symptoms overlapping endophenotypes has made MDD diagnosis and treatment challenging in general practice, necessitating more precise clinical techniques.

Recently, evolutionary molecular biology has coined microRNAs (miRNAs) as master gene expression regulators that have revolutionized molecular medicine [18]. These short-conserved RNA molecules are estimated to regulate up to 60% of all mammalian protein-coding genes [19,20]. Interestingly, 70% of these miRNAs regulate various neuronal processes such as neurogenesis and neuroplasticity [18]. miRNAs are predicted to target either single or multiple mRNAs or act in combination to regulate the expression of multiple mRNAs [18,21]. Any dysregulation of mRNA may result in the abridged translation of overall protein output [19,22]. However, other than their role in protein expression and translation, miRNAs directly impact double-stranded DNA (dsDNA) through hybridization to form triplexes and result in transcriptional gene silencing [19,23]. It is also proposed that miRNAs bind to single-stranded DNA (ssDNA) and influence downstream transcription of target genes through chromatin modifications [19,24]. It is observed that miRNAs are involved in DNA damage repair via miRNA-DNA hybridization [25]. Moreover, miRNA also regulates DNA methylation, and inversely DNA methylation may also influence the miRNA activity [26,27,28,29]. Overall, miRNAs play a significant role in a plethora of biological processes during normal and disease states [30]. Based on miRbase release 22.1 databases, about 2588 mature human miRNAs have been reported so far [31], and 500 miRNAs are found in detectable amounts of blood samples [32]. 

In the past few years, research on circulatory miRNAs (cimiRNAs) as biological indicators of various pathogenic conditions has gained momentum [33]. They travel in bodily fluids such as serum [34], plasma [35], urine [36], cerebrospinal fluid [34], and saliva [37,38] and impart their activity in distant cells or tissues to administer endocrine gene regulation [39]. Notably, several reports have associated dysregulated cimiRNAs with different pathological and psychological conditions, including psychiatric disorders, neurological disorders, cancer, and other metabolic disorders. In comparison to metabolomic [40] and proteomic biomarkers [41], miRNAs propose various advantages over other RNA-based molecules, such as robust stability in bodily fluids [42], specific expression in particular tissues [41], less variation in results between gender, age or time of sampling [43,44], and fast and accurate quantification using routine laboratory methods (RT-qPCR) [45]. Thus, accurate interpretations can be deduced because mature miRNA levels usually align with miRNA activity, whereas post-translational modifications of proteins usually result in complex correlations between protein activity and expression levels. In MDD, several miRNAs have been identified in disease progression by targeting various pathogenic proteins or associated pathways, and they are dysregulated by pathogenic protein expression [46]. Moreover, several reports have presented dysregulation of cimiRNAs in the CSF or blood of MDD patients [47,48]. Therefore, altogether, a minimally invasive screening approach for cimiRNAs in biological samples of MDD may offer a viable tool for specific and optimal diagnosis and therapy response monitoring of this disorder. Henceforth, in this review, we interpreted the results of previous independent studies to probe cimiRNAs as molecular indicators for MDD. Moreover, we performed in-silico analysis to evaluate cimiRNAs for their future diagnostic potential along with suspected perils and proposed pathways, biological processes, and chromosomal distribution analysis to broaden their putative roles in MDD psychopathogenesis.

## 2. Methods

**Figure 1:**
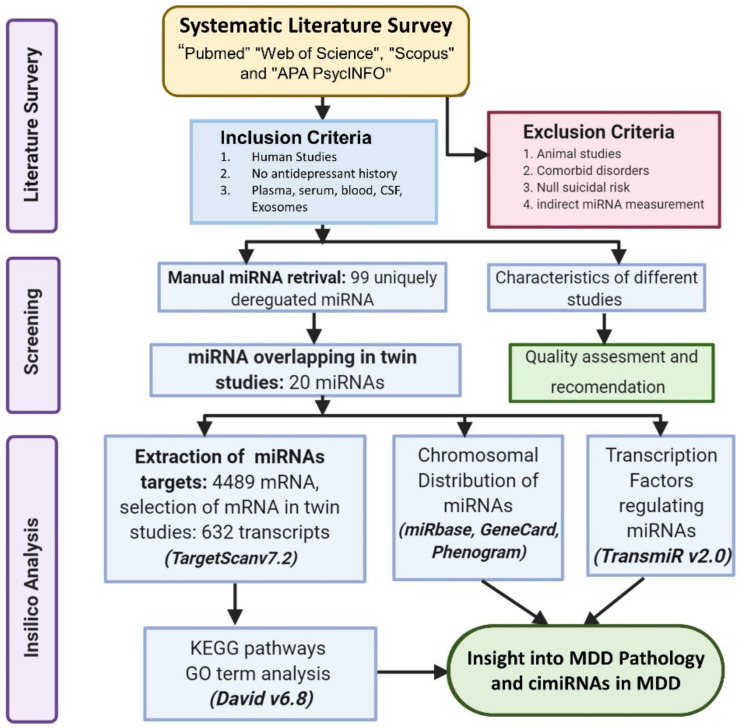
Illustration of the scheme of the study.

This systematic review was designed according to suitable reporting items of PRISMA statements. Prisma flow chart illustrating study selection and PRISMA checklist are available in the Supplementary Materials (Table S1, Figure S1). Overall, the procedure is depicted schematically in Figure 1.

### 2.1. Literature Search and Selection

The literature search was conducted by using Boolean terms TITLE-ABS-KEY (“microRNAs”); (“Major depressive disorder” OR “MDD”) AND (“miRNA”; “Major depressive disorder” OR “MDD”) AND (“Blood” OR “Serum” OR “Plasma” OR “CSF” OR “Exosomes”) in the electronic databases PubMed, Web of Science, Scopus and APA PsycINFO. Each database was searched from its inception (2012) till January 2021. No language limit was applied during the literature search. It yielded 1004 articles with five review articles that were manually searched for more relevant studies. Duplicate records were removed. Three reviewers (MR, RA and SF) independently screened titles and abstracts of all reports obtained by this search. The same authors (MR, RA and SF) reviewed the full texts of selected articles. Disagreements were resolved after discussion with the fourth and fifth authors (NA and AN).

### 2.2. Inclusion and Exclusion Criteria

This study included only those studies that strictly met the inclusion criteria: (i) human studies, (ii) patients with zero histories of antidepressant treatment or resilience, (iii) quantitative screening of cimiRNAs obtained from body fluids (CSF, blood, plasma, and serum) or exosomes. However, some studies such as, (i) animal studies (ii) studies of patients with comorbid disorders such as cardiovascular issues, hypertension, renal problems, stroke, diabetes, alcohol or drug abuse, infectious disease, eating disorders, personality disorders, and epilepsy, (iii) studies of null suicidal risk and (iv) other meta-analyses with no precise miRNA measurement, were excluded. No restriction was applied to the number of participants in this study. The study selection process is summarized in Figure S1.

### 2.3. Data Extraction

Three authors (MD, SF and KMU) independently extracted data from each selected report. Data obtained from qualified studies included types of clinical scales for MDD diagnosis, epidemiology of MDD study, the prevalence of MDD among males and females, cimiRNA sources: exosomes or body fluid type (CSF, blood, serum, plasma), strategies used for cimiRNA detection (qRT-PCR, microarray assays, and NGS), and cimiRNA expression and its role in MDD psychopathophysiology. “cimiRNAs” were manually named circulatory miRNAs in this study according to the name found in mirbase database (http://www.mirbase.org, accessed on 2 November 2020). Studies were compared with data to identify conflicting results (cimiRNA expression) presented by selected reports. Studies with similar cimiRNA expression in two or more reports were included. All extracted data were rechecked and reconfirmed by the fourth author (NA) and then analyzed.

### 2.4. Prediction of cimiRNA Targets and In-Silico Analysis

mRNA targets of cimiRNAs were extracted from TargetScan 7.2 website (http://www.targetscan.org/vert_72, accessed on 22 October 2020) with a context plus score threshold of less than −0.3. Data were compiled into a single list using MS Excel. mRNAs targeted by two or more cimiRNAs were selected for further analysis. In-silico analysis including GO analysis (gene ontology, biological processes, molecular function, and cellular components), KEGG pathway (Kyoto encyclopedia of genes and genomes), and InterPro enrichment analysis were performed using DAVID v6.8 (database for annotation, visualization, and integrated discovery) [49].

### 2.5. Chromosomal Location of cimiRNAs 

Human coordinates of each cimiRNA were recruited from the miRbase release 22.1 website (http://www.mirbase.org accessed on 2 November 2020). A chromosomal ideogram was created to visualize data by uploading coordinates using PhenoGram software [50].

### 2.6. Transcription Factors Regulating the cimiRNAs 

Transcription factors (TFs) situated 300 bp upstream and 100 bp downstream of each cimiRNA transcription start site (TSS) were investigated through the TransmiR v2.0 literature-curated database and experimentally validated TF-miRNA regulations [51].

## 3. Results and Discussion

### 3.1. Profiling of Dysregulated cimiRNAs in MDD

**Figure 2:**
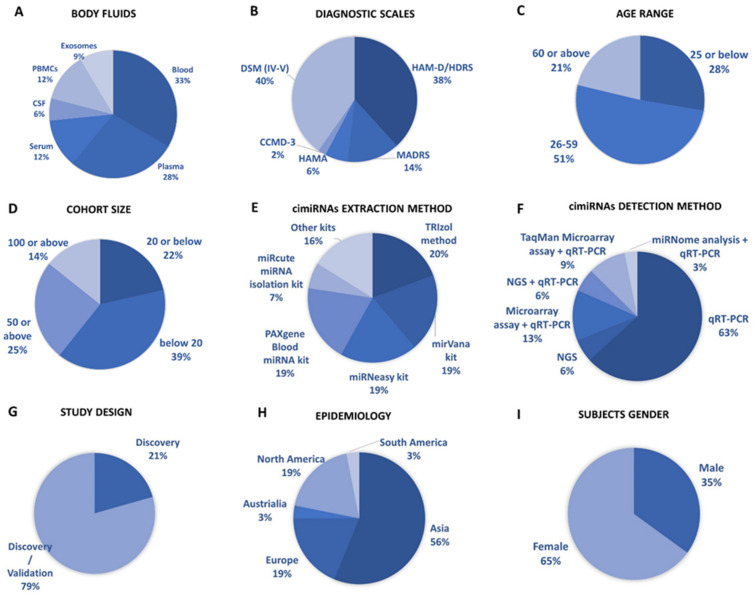
Overview of cimiRNA studies in MDD. (**A**) Fraction of human body fluids analyzed for cimiRNAs in MDD. (**B**) Clinical scales used for MDD diagnosis. (**C**) Age range of participants for MDD studies. (**D**) Number of MDD samples analyzed. (**E**) Extraction strategies employed for cimiRNA isolation. (**F**) Detection strategies used for cimiRNAs. (**G**) Types of studies illustrating either discovery phase or discovery accompanied by a validation phase. (**H**) Epidemiological data collected for depression study from various continents. (**I**) Percentage of males and females reported in depression studies.

**Figure 3:**
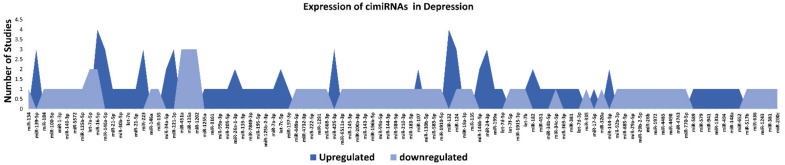
List and number of studies of dysregulated cimiRNAs in MDD.

This systematic review identified 32 reports to ascertain dysregulated cimiRNAs in plasma, serum, blood, CSF, and exosomes in MDD patients; Figure 1 and Supplementary Materials Table S2 show a detailed explanation of the data acquired from the 32 studies that served as the basis for this meta-analysis. These studies included 11 blood, nine plasma, four serum, two CSF + blood, four PBMC, and three exosome reports (Figure 2A). Across all reports, a total of 99 cimiRNAs were found dysregulated in MDD; 20 cimiRNAs (20%) were reported in two or more studies, and 10 cimiRNAs (10%) in three or more studies (Figure 3). Most of these cimiRNAs are highly expressed in the brain tissues and regulate neuronal and MDD-associated processes. 

miR-132 is one of the most enriched miRNAs in the brain that is indispensable for controlling axon growth, neuronal differentiation, migration, plasticity, etc. [52]. Lower expression of miR-132 suppresses BDNF expression, which results in MDD and suicidal behavior [53,54]. miR-124-3p is another significant neuronal miRNA involved in regulating neuronal fate determination, development, neuronal plasticity, etc. [55]. It is highly expressed in the brains of MDD patients [56,57] and is suspected to be a potential therapeutic target for designing novel anti-depressive drugs [58]. The Let-7 family of miRNAs are the most abundant miRNAs in the brain, and their dysregulated expression induces neuronal apoptosis, neuroinflammation, and depressive symptoms in humans [59]. To sum up, these investigations propose that specific dysregulation of brain miRNAs may predict some clinical manifestations observed in MDD patients.

Overall, the expression of 75% of cimiRNAs was found up-regulated in the body fluids of depressive patients (Figure 3, Supplementary Materials Table S2). After ruling out other regulatory mechanisms such as transcription factor activity, histone modifications, methylation etc., it was speculated that most mRNA targets are lowered in MDD. In contrast, mRNA targets of downregulated cimiRNAs (25%) will have higher expression levels. Therefore, it is hypothesized that this disequilibrium of mRNA targets may disturb the balance of total gene expression in brain cells and contribute to the neuropsychiatry of MDD. 

Qualitative analysis of all independent reports presented a limited overlap between them. A specific pattern of dysregulated cimiRNA depression signatures was formed after compiling all cimiRNAs from all 32 reports (Figure 3). Variable inconsistency was observed in these reports due to several factors, such as different methodologies adopted by various studies: diverse study designs, isolation, screening, and quantification techniques, etc. To begin, different subjective clinical scales (DSM-IV-V, HAM-D-HDRS, MADRS, HAMA, and CCMD-3) (Figure 2B) utilized by psychiatrists or physicians in these research studies may have impacted MDD diagnosis. MDD is assessed and diagnosed by predefined parameters that include three or more symptoms. However, this diagnostic approach does not seem appropriate because various patients presenting variable endophenotypes of MDD reported severe suicidal thoughts with just one or two defined symptoms. Furthermore, no prerequisite biological testing has been performed in primary clinical settings so far. Therefore, it is anticipated that it may influence the quality of results regarding the severity of MDD due to dependency on the specific parameters used in the MDD diagnosis. In most studies, body fluid samples from the control group (healthy individuals) and MDD patients for miRNA differential expression study were collected after 6–9 h fasting, whereas in some cases, no defined rule was followed. Similarly, sampling from the control group (healthy individuals) and patients was not of an appropriate match regarding age or sex in a few cases (Figure 2C). In addition, a small cohort size (less than 20 subjects) was a major issue due to bias that lowered the possibility of elucidation, either by statistical methods or dysregulated cimiRNAs (Figure 2D). Moreover, biofluid samples (blood, plasma, serum, and CSF) used in these studies contain differential cimiRNA contents (Figure 2D). For instance, CSF possesses more neuronal cimiRNAs than peripheral blood, whereas serum is enriched in more cimiRNAs than plasma due to the coagulation process. However, a comparison between CSF and serum or plasma neuronal cimiRNAs is also described in the results (Supplementary Materials Table S2) while studying genome-wide cimiRNA expression and adjusting Fisher’s value (*p* = 0.017) for different reports. 

Techniques used for cimiRNA isolation are also another significant factor. Various characteristics of miRNAs such as small size, stable secondary structure, and very low counts in body fluids make their extraction and recovery quite challenging through extraction kits (Figure 2E). Different commercial kits possess different strengths (cons/pros) for cimiRNA isolation concerning yield and preference. Eleven different kits and protocols for cimiRNA extraction were described in 32 studies (Supplementary Materials Table S2). Likewise, strategies for cimiRNA detection (Figure 2F) and isolation also influenced the quality of results. Real-time RT-PCR (qRT-PCR) is considered a gold standard method for specific and sensitive miRNA detection, but it was deployed to test a limited number of identified cimiRNAs, thus leaving a majority of dysregulated cimiRNAs undetected. In 21 studies, qRT-PCR was employed for direct cimiRNA quantification and detection, while four studies analyzed hundreds of cimiRNAs through chip microarray assay and qRT-PCR. In contrast, three studies used a real-time PCR-based array with pre-loaded PCR primers to broaden the comparison between samples. Furthermore, four studies reported next-generation sequencing (NGS), another promising method of qualitative and quantitative miRNA profiling. It identifies novel miRNAs but is often limited in terms of reproducibility and replicability due to technological and software issues. However, only one study screened cimiRNAs through miRNome sequencing and verified cimiRNAs by qRT-PCR. 

The utility of dysregulated cimiRNAs was thoroughly investigated between discriminating subjects with MDD and healthy individuals in all 32 studies. Acute variations were found in diagnostic sensitivity and specificity, i.e., 21% of reports discovered dysregulated cimiRNAs together with MDD, whereas 79% of reports validated cimiRNAs after subsequent investigations (Figure 2G). Nine reports showed that cimiRNAs (Supplementary Materials Table S2) were analyzed by incorporation into receiver operating characteristic curves (ROCs) for their specific and sensitive roles as future biomarkers, whereas in other reports, cimiRNAs were merged into panels for more accuracy. Therefore, in terms of the present studies, one may predict that a panel of cross-validated cimiRNAs could serve as a future diagnostic marker. 

Overall, the prevalence of MDD varies significantly across borders, affecting 6% of the population and increasing lifetime risk by three-fold (15–18%). It is estimated that one in five people go through at least one episode of depression at some stage in their lifetime. Epidemiological data from all 32 studies presented considerable disparity across countries (Figure 2H). Most of the subjects were reported from Asia, including 15 studies from China and individual studies from Taiwan, Iran, Turkey, and Israel. Six studies were reported from Europe (UK, Ireland, Romania, Sweden, Italy, and France), and six studies from North America (four reports from Canada and two from the USA). South America reported one study from Brazil, and Australia reported one study. Although these data include reports from most continents, they are still not enough to predict the exact consequences of MDD. For accurate diagnoses through cimiRNAs, a larger sample size with more populations in various regions should be analyzed.

Furthermore, the gender gap in the prevalence of MDD among males and females is another essential aspect. MDD prevails more frequently in females (usually two times higher) than males (Figure 2I). The data analyzed among male and female subjects present coherent results with other studies. However, this gender gap may be linked to various sex differences in susceptibility to various biological, psychological, and environmental factors involved in regulation on the micro and macro levels.

### 3.2. Profile of Dysregulated cimiRNA Targets in MDD

**Figure 4:**
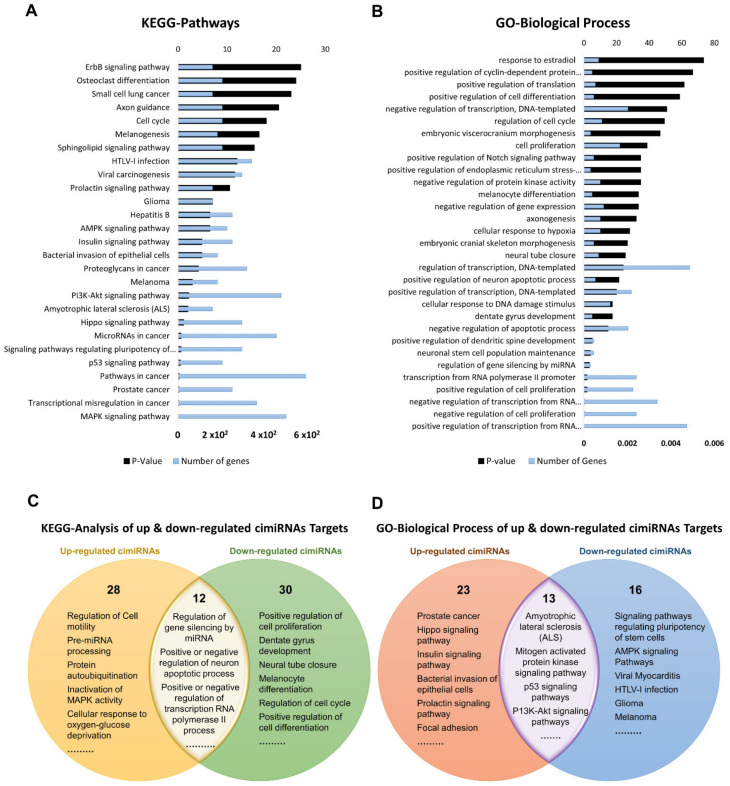
Functional annotation of dysregulated cimiRNA targets regulated by at least two dysregulated cimiRNAs identified in the studies. (**A**) Kyoto Encyclopedia of Gene and Genomes analysis for up- and downregulated targets of cimiRNAs. (**B**) Gene Ontology- Biological Process for up- and downregulated targets of cimiRNAs. (**C**) Venn diagram illustrating comparison of KEGG analysis for up- and downregulated cimiRNA target genes. (**D**) Venn diagram illustrating comparison of Gene Ontology-Biological Process for up- and downregulated cimiRNA target genes.

To discern biological processes and pathways influenced by dysregulated cimiRNAs, DAVID v6.8 was used to analyze the predicted mRNA targets of dysregulated cimiRNAs for KEGG pathways and GO categories (Figure 4). To avoid mistakenly identified cimiRNAs, cimiRNAs found in at least two studies with similar expression (20 out of 99 cimiRNAs, 20%: 4 downregulated, and 16 upregulated) were probed for analysis (Figure 3). However, the criteria for cimiRNA analysis were not made more strict (i.e., referring to cimiRNAs investigated in three or more reports), as this could have limited the number of cimiRNAs to less than 12% of the original cimiRNA list, which may not be suitable for analysis, as most of the studies were not NGS-based. mRNA targets were aligned by screening transcript–miRNA interactions with context plus score threshold less than −0.3, according to the TargetScan 7.2 algorithm. These mRNA targets complied with a list of 4489 transcripts that were further processed to analyze those mRNA transcripts marked by at least two dysregulated cimiRNAs. This second selection further restricted the list of mRNA transcripts to 632 species (14% of the original transcript list). This scrutiny was performed due to the following reasons: (i) most of the previous studies reporting overexpression/knockdown of single miRNAs did not present significant expression, whereas co-expression of two or more miRNAs targeting a specific mRNA ensured enhanced expression; (ii) most neuronal-specific mRNA transcripts are regulated by more than one miRNA collectively, improving mRNA transcript expression and targeting specific transcripts; (iii) false bioinformatic predictions were avoided, i.e., a stringent parameter was set to ensure that at least two cimiRNAs target each mRNAs species. 

Dysregulated expression of cimiRNAs was confirmed twice in the MDD fluid samples with a specific transcript miRNA interaction score to ensure the chances of authentic expression in the MDD. For further validation, we also studied the functional correlation of mRNA transcripts between up-regulated (918 targets) and down-regulated cimiRNAs (270 targets) by overlapping their KEGG and GO items among their targets to predict their role in the psychopathology of MDD. Altogether, 27 KEGG pathways were found, significantly enriched by different dysregulated cimiRNAs (Figure 4A). Overlapped KEGG pathways of up- and down-regulated cimiRNA targets demonstrated various prominent processes such as amyotrophic lateral sclerosis (ALS), mitogen-activated protein kinase (MAPK) signaling pathway, p53 signaling pathways, and P13K-Akt signaling pathways along with other cancer-associated pathways, implicating their role in the pathophysiology of MDD (Figure 4C). Increasing evidence has suggested that MDD causes neuronal damage or neuronal death and is involved in the late onset of amyotrophic lateral sclerosis; for example, miR-34a [60] and miR-24 [61], targeting the BCL-2 (pro-apoptotic protein) are involved in lateral sclerosis, a rare neurological disease [62,63]. Interestingly, two cimiRNAs probed in current neuronal apoptosis could serve as future diagnostic markers.

Among various pathways, the MAPK signaling pathway plays a crucial role in various normal brain functions, including neuronal, synaptic, and structural plasticity, and is vital for neurotrophin/growth factor-mediated neuronal response [64]. This pathway is found in down-regulated cimiRNA (let-7a-5p) analysis as a significant pathway (*p* = 0.0038). Let-7a-5p downregulates RAS expression, suppressing the MAPK signaling pathway, and is positively associated with the depression-like phenotype [65,66]. P13K/Akt signaling cascade is strongly linked with the neurobiology of MDD [67]. Reduced AKT activity was found in several MDD patients [68]. Various cimiRNAs in the current study (miR-144, miR-137, let-7c, let-7b, miR-16, miR-182, miR-223, and miR-451) were found to regulate expression of several genes involved in the P13K/AKT pathway. Regarding MDD-associated mechanisms, lowered TP53 protein (tumor protein p53) expression increased anxiety and depressive-like behavior [69,70]. TP53 protein plays a pivotal role in the regulation of DNA repair, apoptosis, cell cycle progression, and brain development in the early stages. It is proposed to be a potential candidate for neurodegenerative and neuropsychiatric disorders [71,72]. Therefore, based on the current in-silico analysis, it is hypothesized that miR-26a-5p and let-7c targeting TP53 protein are involved in MDD-associated behaviors. Overall, the dysregulated KEGG pathways investigated by this analysis have been associated with MDD.

**Figure 5:**
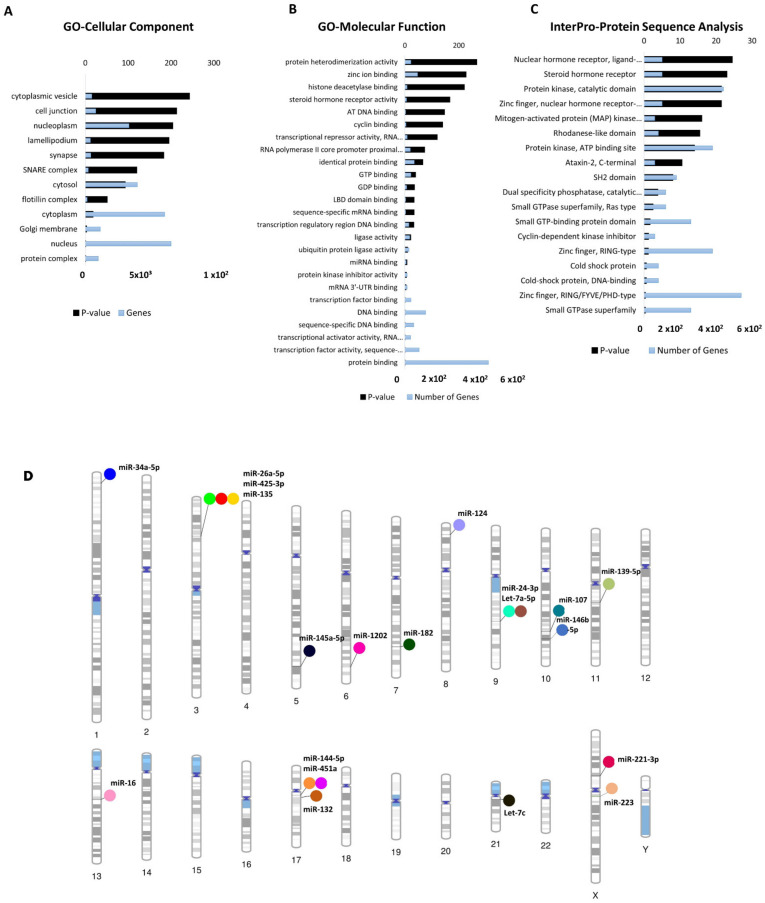
Functional annotation of dysregulated cimiRNA targets and chromosomal distribution of dysregulated cimiRNAs. (**A**) Gene Ontology-Cellular Components of up- and downregulated targets of cimiRNAs. (**B**) Gene Ontology-Molecular Functions of up- and downregulated targets of cimiRNAs. (**C**) InterPro-Protein sequence analysis of up- and downregulated targets of cimiRNAs. (**D**) Phenogram of the chromosomal distribution of dysregulated miRNAs in depression.

Gene ontology (GO) analyses (biological processes (GO-BP), cellular component (GO-CC) and molecular function (GO-MF)) of the dysregulated cimiRNA targets were performed. Most enriched biological processes (BP) (Figure 4B,D) were associated with ‘positive regulation of neuron apoptotic process’ (up miRNAs-7 targets, down miRNAs-5 targets), ‘negative regulation of neuron apoptotic process’ (up miRNAs-43 targets, down miRNAs-11 targets), ‘positive regulation of transcription RNA polymerase II process’ (up miRNAs-60 targets, down miRNAs-21 targets), ‘negative regulation of transcription RNA polymerase II process’ (up miRNAs- 26 targets, down miRNAs-11 targets), ‘axonogenesis’ (up miRNAs-10 targets, down miRNAs-5 targets), ‘cellular response to hypoxia’ (up miRNAs-10 targets, down miRNAs-5 targets) ‘regulation of gene silencing by miRNA’ (up miRNAs-4 targets, down miRNAs-3 targets), etc. Likewise, subcellular compartments (GO-CC) of up- and down-regulated cimiRNAs targets showed that the majority are localized in the nucleus (up miRNAs-190 targets, down miRNAs- 91 targets), nucleoplasm (up miRNAs-96 targets, down miRNAs-44 targets) and cytosol (up miRNAs-113 targets, down miRNAs- 50 targets) (Figure 5A). However, molecular functions (GO-MF) of their targets showed that DNA binding (up miRNAs-74 targets, down miRNAs-38 targets), zinc ion binding (up miRNAs-23 targets, down miRNAs-45 targets), transcription factor activity-specific sequence DNA binding activity (up miRNAs-51 targets, down miRNAs-23 targets), protein binding (up miRNAs-292 targets, down miRNAs-125 targets) and transcription factor binding (up miRNAs-51 targets, down miRNAs-23 targets) are the most enriched categories (Figure 5B). Altogether, the findings from this analysis are rather crucial, as a significant number of target transcripts are involved at transcriptional levels, depicting that cimiRNA dysregulation may disrupt mRNA transcription and involve a second wave of massive regulation, which includes wide aberrations at the transcription level.

In addition, a predicted transcription factor (TF) mRNA target list was analyzed through the David InterPro protein sequence tool to garner a comprehensive understanding of transcription factors (TF) involved in the psychopathology of MDD. This analysis proposed Zinc finger domains (Znf) as the most prominent domains found in transcription factors involved in the MDD (Figure 5C). Literature survey showed that Znf domains are found in the Neur family of neuralized genes with neuralized homology repeat (NHR) domains [73,74]. Two Neur encoding proteins, specifically Neurl1 (neuralized E3 ubiquitin-protein ligase 1) and Neurl1B (neuralized E3 ubiquitin-protein ligase 1), are highly expressed in the brain and mediate various processes, including neuronal development and function through notch signaling [74,75,76,77]. Our analysis showed that Neur protein is targeted by miR-1202 and miR-124 in this study. A study on the adult mouse brain showed that disabling the Znf domain in mNeurl1 downregulated its expression, resulting in impaired hippocampal-dependent memory and synaptic plasticity [75]. Similarly, another study showed that mNeurl1-deficient mice manifested behavioral abnormalities such as ethanol hypersensitivity and olfactory discrimination [78].

Moreover, genome-wide association studies (GWAS) also presented the involvement of Neurl1B along with other genes in the development of various neuropsychiatric disorders [77,79,80], thus, suggesting that Znf domain dysfunction by these miRNAs may cause wide transcription changes that contribute to the neuropsychology of MDD. 

Furthermore, MYCBP2, an E3-ubiquitin ligase MYC binding protein-2, was found to be targeted by miR-16, let-7c and miR-132 in this study. MYCBP2 is expressed in abundance in the brain [81,82] and modulates neuronal growth, synaptogenesis, and synaptic plasticity by mediating various signaling pathways, including p38 MAPK signaling cascades [81]. Alternatively, low expression of MYCBP2 resulted in elevated cAMP-synthesis, which causes neuronal excitability through PKA- and CREB-mediated aberrations in gene expression, thus leading to nociceptive behavior [83], which is associated with anxiety and depressive behavior in later stages [84,85]. Overall, this analysis shows that the mRNA transcripts of these cimiRNAs target various transcription factor proteins that play a significant role in the second wave of transcriptional changes in MDD progression and can be used as future diagnostic biomarkers.

### 3.3. Chromosomal Distribution of Dysregulated cimiRNAs in MDD

Investigating the gene location of dysregulated cimiRNAs helps us gain more insight into their putative role in gene expression and their relationship with chromosomal anomalies previously associated with these regions. To shed more light, chromosomal coordinates of the 20 dysregulated cimiRNAs were obtained from miRbase (release 22.1) and Gene cards (The Human gene database) and visualized using Phenogram (Figure 5D). Surprisingly, a few cimiRNAs were clustered in close proximity, such as miR-26a-5p, miR135a, and miR-425-3p (3p22-p21), miR-24-3p and let 7a-5p (9q22.32), and miR-451a and miR-144-5p (17q11.2), suggesting that they might be co-regulated by specific transcription factors or methylation. A literature survey for potential vulnerabilities linked with these chromosomal regions revealed various gene expression changes that might accelerate depressive-like behaviors. For instance, two SNPs (-45 C/T and -196 G/A) were found in the *CCK* gene promoter region (3p22-p21) in patients with depressive and suicidal behavior [86]. This CCK encodes a neurotransmitter termed cholecystokinin, which is reported to be expressed at elevated levels in MDD and patients with suicidal intentions [87].

Similarly, the *PTCH1* gene (9q22.32), which encodes the patched transmembrane protein, the principal receptor of Sonic hedgehog (Shh) signaling, plays a significant role in neuronal development and patterning hippocampus neurogenesis [88,89]. A study of heterozygous mice (*Ptch1^+/−^*) showed aberrant hippocampal morphogenesis with failed or altered neurogenesis. This dysregulated *PTCH1* gene expression leads to phenotypic manifestations of neuropsychiatric disorders, including anxiety and MDD [90,91]. A significant number of genetic variants were observed in the promoter region, 5HHTLPR (5-hydroxytraptamine transporter-linked polymorphic region) of the *SLC6A4* gene with cytogenic position 17q11.2 [92]. *SLC6A4* encodes solute carrier family 6 member (serotonin transporter), whose abnormal expression levels are predominately associated with various episodes of depression [92]. The genetic mutations in 5HHTLPR are usually linked with the presence of “L allele” (16 GC-rich repeated elements of 20–30 bp) or absence of “S-allele” (14 repeated units of 20–30 bp except for deleted regions from 6th to 8th repeated elements) along with other rare variants containing 15, 19 and >20 repeats and SNPs [93,94,95,96]. Therefore, it is hypothesized that polymorphism or silencing of the *SLC6A4* gene may result in abnormal expression of the *SLC6A4* mRNA, which induces abnormal serotonin uptake and manifests mood disabilities [96,97]. Interestingly, through this analysis, we observed that cimiRNAs clustering on different chromosomes host only those protein-coding genes that are exclusively involved in MDD, thus identifying them as ideal cimiRNA biomarkers for monitoring the progression of MDD in future clinical trials.

### 3.4. Transcription Factors Regulating the Expression of Dysregulated cimiRNAs in MDD

**Figure 6:**
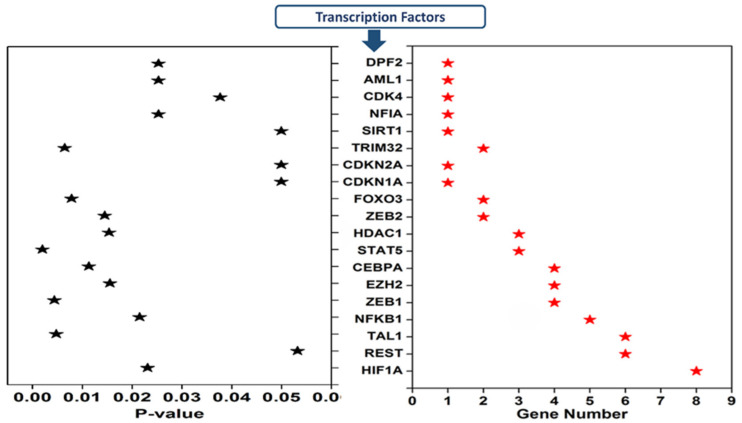
Transcription factors that regulate depression-associated cimiRNAs.

Transcription factors regulating 20 cimiRNAs were investigated through the TransmiR v 2.0 experimental database to analyze TF sites 300 bp upstream and 100 bp downstream of each cimiRNA transcription site. Surprisingly, it was found that TF binding sites for hypoxia-inducible factor-1 (HIF-1), RE1 silencing transcription factor (REST), and T-cell acute lymphocytic leukemia protein 1 (TAL1) were found in most of the cimiRNAs, including cimiRNA clusters located at Chr-3 (miR-26a-5p, miR135a and miR-425-3p) and Chr-9 (miR-24-3p and let 7a-5p). Additionally, nuclear factor kappa B subunit 1 (NFKB1) and zinc finger E-box binding homeobox 1 (ZEB1) were also targeted by most of the cimiRNAs (Figure 6). Thus, to identify whether TFs with four or more miRNAs could be involved in MDD pathogenesis, transcriptomics expression data were thoroughly studied against their targets in MDD patients with healthy controls [98,99]. Accumulating shreds of evidence have shown that abnormal expression of HIF-1 and its target genes, including *CCK,* are profoundly associated with adult neurogenesis defects that trigger MDD pathophysiology [100,101,102]. Further, it is reported that transcriptional factor REST and its targets are also involved in the underlying pathology of MDD through deregulating neuronal function [102,103]. 

Moreover, Samaan et al., in 2015 reported that the obesity risk allele (rs2984618) in the *TAL1* gene is strongly linked with a higher risk of MDD symptoms [103]. Therefore, in view of this analysis and the correlations of miRNAs and target genes with MDD, it is worth mentioning that somatic mosaicism in the genome of brain cells due to inherited genomic instability or epigenetic regulators, especially methylation, may not only deregulate miRNAs targeting transcription factors but also deregulate various genes that lead to the complex etiology of MDD. However, this domain has not yet been studied thoroughly and demands further investigation to understand the underlying neurobiology of MDD.

## 4. Concluding Remarks

The use of cimiRNAs as biomarkers has become a rapidly growing subject owing to their potential to be effective biomarkers for diagnosing malignancies (particularly cancer, diabetes, and cardiovascular disorders). Nonetheless, existing cimiRNAs do not meet the specificity and sensitivity criteria for clinical biomarkers yet, and thus have not been employed in clinical trials so far. cimiRNAs have recently been investigated in CNS diseases and psychiatric disorders, where they can be beneficial biomarkers at an early stage. As discussed in this study, cimiRNAs may aid in deciphering the complicated pathophysiological pathways of MDD. However, cimiRNAs are still in their infancy as valid biomarkers for MDD and other diseases. Variable factors contribute to or influence cimiRNA expression levels. This systematic review showed that various reports contained inconsistencies and/or contradictions about cimiRNA expression levels. It is speculated that these variations may have been caused by technical and methodological variances regarding sample types, collection, processing, detection strategy, RNA extraction, and options for additional downstream statistical analysis. Among various factors, the types of body fluid, such as CSF, blood, and blood cell-free samples, that are utilized to measure cimiRNAs are crucial, since there are discrepancies in cimiRNA expression directions. Further, age, lifestyle, dietary pattern, and ethnicity might also impact cimiRNA expression in MDD. Therefore, diversified control groups focused on single blood compartments would be required to boost the specificity of putative diagnostic biomarkers for MDD. The later technique, on the other hand, may prove difficult in comparing different research studies for review. Additionally, methodological and technical considerations such as sequencing platform (NGS vs. RT-qPCR), sample collection, purification, and data normalization should be taken into account. 

In the second part of the review, we attempted to identify commonly dysregulated cimiRNAs in two or more reports verified by ROC curves, NGS, and qRT-PCRs, and performed in-silico analysis. This in-silico analysis showed that MDD-dysregulated cimiRNAs target many transcription factors, thus suggesting that following the first wave of cimiRNA regulation of expression, the second wave of transcription-wide alterations may further change the proteome of MDD-affected cells. Furthermore, pairing or clustering of some cimiRNAs in some chromosomal positions depicted their coordinated dysregulation due to specific transcriptional activity or methylation. Additional analysis demonstrated that they specifically target those genes primarily involved in the MDD-associated mechanisms. Still, all of these predicted findings are constrained by experimental validation for target specificity, thus requiring more investigation to shed more light on the significance of these bioinformatics insights. In conclusion, this study identified various obstacles for cimiRNAs as MDD biomarkers and applied in-silico analysis to commonly dysregulated cimiRNAs in multiple reports to envision the underlying molecular psychopathology of MDD, which may aid researchers in using experimental validation to imply their roles in MDD development. Increasing research interest in cimiRNAs and their putative roles in MDD psychopathogenesis shows that this field can evolve further, and cimiRNAs may have the capacity to detect MDD in the near future. However, technological and scientific techniques must be improved before cimiRNAs can be used as clinical biomarkers.

## Data Availability

All supporting data generated and analyzed during this study are included within the manuscript (and its Supplementary Materials).

## Supplementary Materials

The following supporting information can be downloaded at: https://www.mdpi.com/article/10.3390/ijms23031294/s1.

## Abbreviations

MDD: Major Depressive Disorder; cimiRNAs, Circulatory miRNAs; CSF, Cerebrospinal fluid; PBMCs, Peripheral Blood Mononuclear Cells; DSM-IV-V, Diagnostic and Statistical Manual of Mental Disorders fourth and fifth edition; MADRS-S, Montgomery Asberg Depression Scale; HAM-D/HDRS, Hamilton Rating Scale for Depression, HAMA, Hamilton Rating Scale for Anxiety; CCMD-3, Chinese Classification of Mental Disorders; fMRI, Functional Magnetic Resonance Imaging; ALS, Amyotrophic Lateral Sclerosis; MAPK, Mitogen-Activated Protein Kinase; TF, Transcription Factor.
