# Supplementary material for: A Systematic Review of Circulatory microRNAs in Major Depressive Disorder: Potential Biomarkers for Disease Prognosis
Source: Int J Mol Sci. 2022 Jan 24;23(3):1294. doi: 10.3390/ijms23031294 (PMC8835958; doi:10.3390/ijms23031294)
Supplement: Supplementary file 1 [file ijms-23-01294-s001.zip › Supplementary Table S2.pdf]

Supplementary Table S2: Data from 32 studies of cimiRNAs in depression

S.No	Reference	miRNA (Up)	miRNA (Down)	Biofluid	Country	ROC	Validation	Diagnostic Criteria (Severity)	Depression Subjects	RNA Extraction Method	Mirna Detection Strategy	Reference Gene	Male	Female	Age
1	(Liang et al., 2020)	miR-139-5p		Serum (exosome)	china	YES	miR-139-5p	HAMD, DSM-V, MADRS	30/30	TRIzol reagent	RT-qPCR	miR-16	20	10	32.6± 10.87
2	(H. Zhang et al., 2020)		miR-134	plasma	china	YES	miR-134	HAM-D	100/100	TRIzol reagent	RT-qPCR	U6	37	63	20-40
3	(Wei et al., 2020)	miR-139-5p		Blood derived exosomes	china	YES	miR-139-5p	HAMD, DSM-IV, MADRS	33	TRIzol reagent	RT-qPCR	miR-16			
4	(Mendes-silva et al., 2019)		miR-184, miR-100-5p, miR-1-3p, miR-140-3p, miR-5572, miR-125b-5p, let-7a-5p	Plasma	Brazil	YES	miR-184	DSM-V, HDRS-21	63/53	mirVana PARIS kit	NGS + qPCR	U6	11	52	60-80
5	(Kyunghee et al., 2019)	miR-16-5p, miR-146a-5p, miR-21-5p, miR-30b-5p		Plasma	Canada			Not reported	Not reported	mirVana miRNA isolation kit	NGS	Not reported	Not reported	Not reported	42-50
6	(Hung et al., 2019)	let-7e, miR-21-5p,	miR-146a, miR-155	PBMC (whole blood)	Taiwan		miR-146a, miR-155, let-7e	DSM-V, HAMD	84/43	Quick-RNA MiniPrep kits	RT-qPCR	U6	20	64	20-65

		miR-223													
7	(Kuang et al., 2019)	miR-34a-5p, miR-221-3p	miR-451a	serum	china		validated	DSM-IV, HAMD	84/78	Qaigen kit (USA)	qPCR	U6	30	54	35.51-13.62
8	(Gheysarzadeh et al., 2018)		miR-16, miR-135a, miR-1202	serum	Iran	YES	validated	DSM-IV	39/36	TRIzol reagent	qPCR	U6	18	21	20-40
9	(Y. Zhang et al., 2018)	miR-1255a, miR-3161, miR-99a-3p, miR-205-5p, miR-26a-1-3p, miR-139-5p, miR-7849-3p, miR-195-5p, miR-125b-2-3p, miR-7ca-3p, let-7c-5p, miR-197-3p	miR-449a-5p, miR-4732-3p, miR-222-5p, miR-1291, miR-668-3p, miR-425-3p, miR-6511a-3p, miR-145-3p, miR-200a-3p, miR-143-3p, miR-196b-5p, miR-99a-5p, miR-144-3p, miR-584-5p, miR-210-3p, miR-183-5p, miR-107, miR-130b-5p, miR-589-	Serum (exosome)	china		discovery	MADRS-S		ExoRNeasy serum/plasma kit	NGS				20-64

			5p, miR-1910-5p												
10	(Fang et al., 2018)	miR-132, miR-124		Plasma	china		validation	HAMD, HAMA	45/32	miRcute miRNA isolation kit	qPCR	5S rRNA	19	26	40
11	(Marques et al., 2017)		miR-19a-3p	plasma	Australia		miR-19a-3p	DSM-IV	32/55	miRNeasy serum/plasma kit (qaigen)	qPCR	miR-17-5p	32		40
12	(Fiori et al., 2017)	miR-16	miR-1202, miR-135	Whole blood	Canada	YES	validation	HAMD, MADRS	200	miRNeasy serum/plasma kit (qaigen), PAXgene Blood miRNA Kit (Qiagen, Canada)	qPCR				
13	(Lopez et al., 2017)	miR-146a-5p, miR-146b-5p, miR-24-3p, miR-425-3p,		BLOOD/PLASMA	EUROPE		Discovery and validation	MADRS	319	PAXgene Blood miRNA Kit (Qiagen, Canada)	NGS+ FlexArray package (microarray) + TaqMan RT-PCR microRNA assay	RNU6B	161	326	19-74
14	(Roy et al., 2017)	miR-124		serum	USA		validation	MADRS	18/17	TRIzol reagent	qPCR	U6			
15	(Maffioletti et al., 2016)	miR-199a, miR-24-3p, miR-425-3p	let-7a-5p, let-7d-5p, let-7f-5p, miR-1915-3p	blood	Italy-UK		miR-24-3p, miR-425-3p, let-7a-5p, let-7d-5p, let-7f-5p	DSM-IV HAMD, HAMA	20/20	PAXGene Blood miRNA Kit	TaqMan Microarray assay, RT-qRCR	U6	3	17	24-40.7

16	(Gururajan et al., 2016)	let-7b, let-7c, miR-16, miR-182, miR-451, miR-223		blood	Ireland		let-7b, let-7c	HDRS	40/20	PAXGene Blood miRNA Kit	TaqMan Microarray assay, RT-qRCR	-			35-42
17	(Liu et al., 2016)	miR-132		blood	China		miR-132	DSM-IV HAMD, HAMA	62/73	MagMAX RNA Isolation Kit	RT-qRCR	U6	22	40	20-36
18	(He et al., 2016)	miR-124		PBMCs	china	YES	miR-124	DSM-IV HDRS	32/30	mirVana TM miRNA Isolation	RT-qRCR	U6	12	30	24-44
19	(Sun et al., 2016)	miR-34b-5p, miR-34c-5p, miR-369-3p, miR-381, miR-107		PBMCs	china		miR-34b-5p, miR-34c-5p	DSM-IV,	32/30	TRIzol reagent	RT-qRCR	U6	13	19	35-45
20	(Enatescu et al., 2016)	miR-146a-5p, miR-146b-5p, miR-221-3p, miR-24-3p, miR-26a-5p		Plasma	Romania			DSM-IV, HDRS, HAMD		miRNeasy Serum/Plasma kit	Microarray assay, qRT-RCR				18-65
21	(Wan et al., 2015)	miR-221-3p,	miR-451a	CSF, Blood	china	YES	miR-221-3p, miR-	DSM-IV, HDRS	38/27	TRIzol reagent	qRT-RCR		18	20	24-45

		miR-34a-5p, let-7d-3p					34a-5p, let-7d-3p, miR-451a								
22	(Su et al., 2015)	miR-132		blood	China		miR-132		30/30	kit	qRT-RCR	U6			
23	(J. Li et al., 2015)		miR-335	blood	China		miR-335	DSM-IV, HAMD	18/18	miRNeasy Mini Kit	qRT-RCR	U6	8	10	20-30
24	(Camkurt et al., 2015)	miR-451a, miR-17-5p, miR-223-3p	miR-320a,	plasma	Turkey		miR-451a, miR-320a,	DSM-IV, HDRS, MADRS	50/41	High Pure miRNA Isolation Kit	qRT-RCR		15	35	25-50
25	(Wang et al., 2015)		miR-144-5p, miR-92b-3p, miR-885-5p, miR-30a-5p, miR-29a-5p, miR-29b-2-5p	plasma	Sweden		miR-144-5p	HADS-A, HADS-D, MADRS	169/52	miRNeasy Mini Ki	qRT-RCR		13	86	20-64
26	(Song et al., 2015)		miR-16	CSF, Blood	china		miR-16	HAMD	36/30	miRcute miRNA Isolation Kit	qRT-RCR	U6	15	21	18-45
27	(Lopez et al., 2014)		miR-1202	blood	Canada		miR-1202	DSM-IV	50/29	miRNeasy Mini Kit	Microarray assay, qRT-RCR	RNU6B			
28	(Issler et al., 2014)		miR-135	blood	Israel and USA		miR-135	HDRS	12/12	PAXgene Blood RNA Kit	qRT-RCR				
29	(Fan et al., 2014)		miR-26b, miR-1972, miR-4485, miR-4498, miR-4743	PBMC	China	YES	miR-26b, miR-1972, miR-4485, miR-4498, miR-4743	DSM-IV	81/46	mirVana kit	Microarray assay, qRT-RCR	RNU48	35	46	27-30

30	(Y. J. Li et al., 2013)	miR-132, miR-182		Serum	China		miR-132, miR-182	DSM-IV, CCMD-3	40/40	mirVanaTM miRNA isolation kit	qRT-RCR	5SrRNA	20	20	23-61
31	(Bocchio-Chiavetto et al., 2013)		miR-34c-5p, miR-770-5p	blood	Italy			DSM-IV, HAMD	10/10	PAXgene Blood RNA Kit	miRNome analysis, qRT-RCR	U44 and U48	5	5	18-65
32	(Belzeaux et al., 2012)	miR-589, miR-579, miR-941, miR-133a, miR-494, miR-107, miR-148a, miR-652, miR-425-3p	miR-517b, miR-636, miR-1243, miR-381, miR-200c	blood	France			DSM-IV, HDRS	16-13	mirVana miRNA isolation kit	Microarray assay, qRT-RCR		9	7	35-75

References

1. Belzeaux, R., Bergon, A., Jeanjean, V., Loriod, B., Formisano-Tréziny, C., Verrier, L., Loundou, A., Baumstarck-Barrau, K., Boyer, L., Gall, V., Gabert, J., Nguyen, C., Azorin, J. M., Naudin, J., & Ibrahim, E. C. (2012). Responder and nonresponder patients exhibit different peripheral transcriptional signatures during major depressive episode. *Translational Psychiatry*, *2*(September). https://doi.org/10.1038/tp.2012.112
2. Bocchio-Chiavetto, L., Maffioletti, E., Bettinsoli, P., Giovannini, C., Bignotti, S., Tardito, D., Corrada, D., Milanesi, L., & Gennarelli, M. (2013). Blood microRNA changes in depressed patients during antidepressant treatment. *European Neuropsychopharmacology*, *23*(7), 602–611. https://doi.org/10.1016/j.euroneuro.2012.06.013
3. Camkurt, M. A., Acar, Ş., Coşkun, S., Güneş, M., Güneş, S., Yilmaz, M. F., Görür, A., & Tamer, L. (2015). Comparison of plasma MicroRNA levels in drug naive, first episode depressed patients and healthy controls. *Journal of Psychiatric Research*, *69*, 67–71. https://doi.org/10.1016/j.jpsychires.2015.07.023

4. Enatescu, V. R., Papava, I., Enatescu, I., Antonescu, M., Anghel, A., Seclaman, E., Sirbu, I. O., & Marian, C. (2016). Circulating plasma micro RNAs in patients with major depressive disorder treated with antidepressants: A pilot study. *Psychiatry Investigation*, *13*(5), 549–557. https://doi.org/10.4306/pi.2016.13.5.549
5. Fan, H. min, Sun, X. Y., Guo, W., Zhong, A. F., Niu, W., Zhao, L., Dai, Y. H., Guo, Z. M., Zhang, L. Y., & Lu, J. (2014). Differential expression of microRNA in peripheral blood mononuclear cells as specific biomarker for major depressive disorder patients. *Journal of Psychiatric Research*, *59*, 45–52. https://doi.org/10.1016/j.jpsychires.2014.08.007
6. Fang, Y., Qiu, Q., Zhang, S., Sun, L., Li, G., Xiao, S., & Li, X. (2018). Changes in miRNA-132 and miR-124 levels in non-treated and citalopram-treated patients with depression. *Journal of Affective Disorders*, *227*, 745–751. https://doi.org/10.1016/j.jad.2017.11.090
7. Fiori, L. M., Lopez, J. P., Richard-Devantoy, S., Berlim, M., Chachamovich, E., Jollant, F., Foster, J., Rotzinger, S., Kennedy, S. H., & Turecki, G. (2017). Investigation of miR-1202, miR-135a, and miR-16 in Major Depressive Disorder and Antidepressant Response. *International Journal of Neuropsychopharmacology*, *20*(8), 619–623. https://doi.org/10.1093/ijnp/pyx034
8. Gheysarzadeh, A., Sadeghifard, N., Afraidooni, L., Pooyan, F., Mofid, M., Valadbeigi, H., Bakhtiari, H., & Keikhavani, S. (2018). Serum-based microRNA biomarkers for major depression: MiR-16, miR-135a, and miR-1202. *Journal of Research in Medical Sciences*, *23*(1), 69. https://doi.org/10.4103/jrms.jrms_879_17
9. Gururajan, A., Naughton, M. E., Scott, K. A., O'Connor, R. M., Moloney, G., Clarke, G., Dowling, J., Walsh, A., Ismail, M. F., Shorten, G., Scott, L., McLoughlin, D. M., Cryan, J. F., & Dinan, T. G. (2016). MicroRNAs as biomarkers for major depression: A role for let-7b and let-7c. *Translational Psychiatry*, *6*(8), e862–e862. https://doi.org/10.1038/tp.2016.131
10. He, S., Liu, X., Jiang, K., Peng, D., Hong, W., Fang, Y., Qian, Y., Yu, S., & Li, H. (2016). Alterations of microRNA-124 expression in peripheral blood mononuclear cells in pre- and post-treatment patients with major depressive disorder. *Journal of Psychiatric Research*, *78*, 65–71. https://doi.org/10.1016/j.jpsychires.2016.03.015
11. Hung, Y., Wu, M., Tsai, M., & Huang, Y. (2019). *and miR-155 Correlates with Severity of Depression in Patients with Major Depressive Disorder and Is*. *Mdd*, 1–12.
12. Issler, O., Haramati, S., Paul, E. D., Maeno, H., Navon, I., Zwang, R., Gil, S., Mayberg, H. S., Dunlop, B. W., Menke, A., Awatramani, R., Binder, E. B., Deneris, E. S., Lowry, C. A., & Chen, A. (2014). MicroRNA 135 is essential for chronic stress resiliency, antidepressant efficacy, and intact serotonergic activity. *Neuron*, *83*(2), 344–360. https://doi.org/10.1016/j.neuron.2014.05.042
13. Kuang, W.-H., Dong, Z.-Q., Tian, L.-T., & Li, J. (n.d.). *MicroRNA-451a, microRNA-34a-5p, and microRNA-221-3p as predictors of response to antidepressant treatment*. https://doi.org/10.1590/1414-431X20187212
14. Kyunghee, H., Tyryshkin, K., Elmi, N., Dharsee, M., Evans, K. R., Good, J., Javadi, M., Mccormack, S., Vaccarino, A. L., Zhang, X., Cristina, A., & Feilotter, H. (2019). Plasma microRNA expression levels and their targeted pathways in patients with major depressive disorder who are responsive to duloxetine treatment. *Journal of Psychiatric Research*, *110*(December 2018), 38–44. https://doi.org/10.1016/j.jpsychires.2018.12.007
15. Li, J., Meng, H., Cao, W., & Qiu, T. (2015). MiR-335 is involved in major depression disorder and antidepressant treatment through targeting GRM4. *Neuroscience Letters*, *606*, 167–172. https://doi.org/10.1016/j.neulet.2015.08.038
16. Li, Y. J., Xu, M., Gao, Z. H., Wang, Y. Q., Yue, Z., Zhang, Y. X., Li, X. X., Zhang, C., Xie, S. Y., & Wang, P. Y. (2013). Alterations of Serum Levels of BDNF-Related miRNAs in Patients with Depression. *PLoS ONE*, *8*(5), 1–7. https://doi.org/10.1371/journal.pone.0063648
17. Liang, J. Q., Liao, H. R., Xu, C. X., Li, X. L., Wei, Z. X., Xie, G. J., & Cheng, Y. (2020). Serum exosome-derived miR-139-5p as a potential biomarker for major depressive disorder. *Neuropsychiatric Disease and Treatment*, *16*, 2689–2693. https://doi.org/10.2147/NDT.S277392
18. Liu, Y., Yang, X., Zhao, L., Zhang, J., Li, T., & Ma, X. (2016). Increased miR-132 level is associated with visual memory dysfunction in patients with depression. *Neuropsychiatric Disease and Treatment*, *Volume 12*, 2905–2911. https://doi.org/10.2147/NDT.S116287

19. Lopez, J. P., Fiori, L. M., Cruceanu, C., Lin, R., Labonte, B., Cates, H. M., Heller, E. A., Vialou, V., Ku, S. M., Gerald, C., Han, M. H., Foster, J., Frey, B. N., Soares, C. N., Müller, D. J., Farzan, F., Leri, F., Macqueen, G. M., Feilotter, H., … Turecki, G. (2017). MicroRNAs 146a/b-5 and 425-3p and 24-3p are markers of antidepressant response and regulate MAPK/Wnt-system genes. *Nature Communications*, *8*(May), 1–12. https://doi.org/10.1038/ncomms15497
20. Lopez, J. P., Lim, R., Cruceanu, C., Crapper, L., Fasano, C., Labonte, B., Maussion, G., Yang, J. P., Yerko, V., Vigneault, E., El Mestikawy, S., Mechawar, N., Pavlidis, P., & Turecki, G. (2014). MiR-1202 is a primate-specific and brain-enriched microRNA involved in major depression and antidepressant treatment. *Nature Medicine*, *20*(7), 764–768. https://doi.org/10.1038/nm.3582
21. Maffioletti, E., Cattaneo, A., Rosso, G., Maina, G., Maj, C., Gennarelli, M., Tardito, D., & Bocchio-Chiavetto, L. (2016). Peripheral whole blood microRNA alterations in major depression and bipolar disorder. In *Journal of Affective Disorders* (Vol. 200). Elsevier. https://doi.org/10.1016/j.jad.2016.04.021
22. Marques, F. Z., Eikelis, N., Bayles, R. G., Lambert, E. A., Straznicky, N. E., Hering, D., Esler, M. D., Head, G. A., Barton, D. A., Schlaich, M. P., & Lambert, G. W. (2017). A polymorphism in the norepinephrine transporter gene is associated with affective and cardiovascular disease through a microRNA mechanism. *Molecular Psychiatry*, *22*(1), 134–141. https://doi.org/10.1038/mp.2016.40
23. Mendes-silva, A. P., Tiemi, P., Regina, J., Lucio, A., Marciano, E., Henrique, P., Guedes, G., Scarabeli, L., Barroso, S., Souza, M. De, Diniz, J., Ferreira, R., Bertola, L., Souza, E. De, Tolentino-araújo, G. T., Moreira, C., Ferreira, S., Sabrina, T., Diniz, L., … Satler, B. (2019). Brain-enriched MicroRNA-184 is downregulated in older adults with major depressive disorder : A translational study. *Journal of Psychiatric Research*, *111*(July 2018), 110–120. https://doi.org/10.1016/j.jpsychires.2019.01.019
24. Roy, B., Dunbar, M., Shelton, R. C., & Dwivedi, Y. (2017). Identification of MicroRNA-124-3p as a Putative Epigenetic Signature of Major Depressive Disorder. *Neuropsychopharmacology*, *42*(4), 864–875. https://doi.org/10.1038/npp.2016.175
25. Song, M. F., Dong, J. Z., Wang, Y. W., He, J., Ju, X., Zhang, L., Zhang, Y. H., Shi, J. F., & Lv, Y. Y. (2015). CSF miR-16 is decreased in major depression patients and its neutralization in rats induces depression-like behaviors via a serotonin transmitter system. *Journal of Affective Disorders*, *178*, 25–31. https://doi.org/10.1016/j.jad.2015.02.022
26. Su, M., Hong, J., Zhao, Y., Liu, S., & Xue, X. (2015). MeCP2 controls hippocampal brain-derived neurotrophic factor expression via homeostatic interactions with microRNA-132 in rats with depression. *Molecular Medicine Reports*, *12*(4), 5399–5406. https://doi.org/10.3892/mmr.2015.4104
27. Sun, N., Lei, L., Wang, Y., Yang, C., Liu, Z., Li, X., & Zhang, K. (2016). Preliminary comparison of plasma notch-associated microRNA-34b and -34c levels in drug naive, first episode depressed patients and healthy controls. *Journal of Affective Disorders*, *194*, 109–114. https://doi.org/10.1016/j.jad.2016.01.017
28. Wan, Y., Liu, Y., Wang, X., Wu, J., Liu, K., Zhou, J., Liu, L., & Zhang, C. (2015). Identification of differential microRNAs in cerebrospinal fluid and serum of patients with major depressive disorder. *PLoS ONE*, *10*(3), 1–12. https://doi.org/10.1371/journal.pone.0121975
29. Wang, X., Sundquist, K., Hedelius, A., Palmér, K., Memon, A. A., & Sundquist, J. (2015). Circulating microRNA-144-5p is associated with depressive disorders. *Clinical Epigenetics*, *7*(1), 1–8. https://doi.org/10.1186/s13148-015-0099-8
30. Wei, Z., Xie, G., Mao, X., Zou, X., Liao, Y., Liu, Q., Wang, H., & Cheng, Y. (2020). Exosomes from patients with major depression cause depressive-like behaviors in mice with involvement of miR- 139-5p-regulated neurogenesis. *Neuropsychopharmacology*, *January*. https://doi.org/10.1038/s41386-020-0622-2
31. Zhang, H., Liu, X., Chen, J., Cheng, K., Bai, S., Zheng, P., Zhou, C., Wang, W., Wang, H., Zhong, L., & Xie, P. (2020). Circulating microRNA 134 sheds light on the diagnosis of major depressive disorder. *Translational Psychiatry*. https://doi.org/10.1038/s41398-020-0773-2
32. Zhang, Y., Zhao, Y., Tian, C., Wang, J., Li, W., & Zhong, C. (2018). Differential exosomal microRNA profile in the serum of a patient with depression. *European Journal of Psychiatry*, *32*(3), 105–112. https://doi.org/10.1016/j.ejpsy.2017.10.002
