# Supplementary figures and images for: A Systematic Review of Circulatory microRNAs in Major Depressive Disorder: Potential Biomarkers for Disease Prognosis
Source: Int J Mol Sci. 2022 Jan 24;23(3):1294. doi: 10.3390/ijms23031294 (PMC8835958; doi:10.3390/ijms23031294)

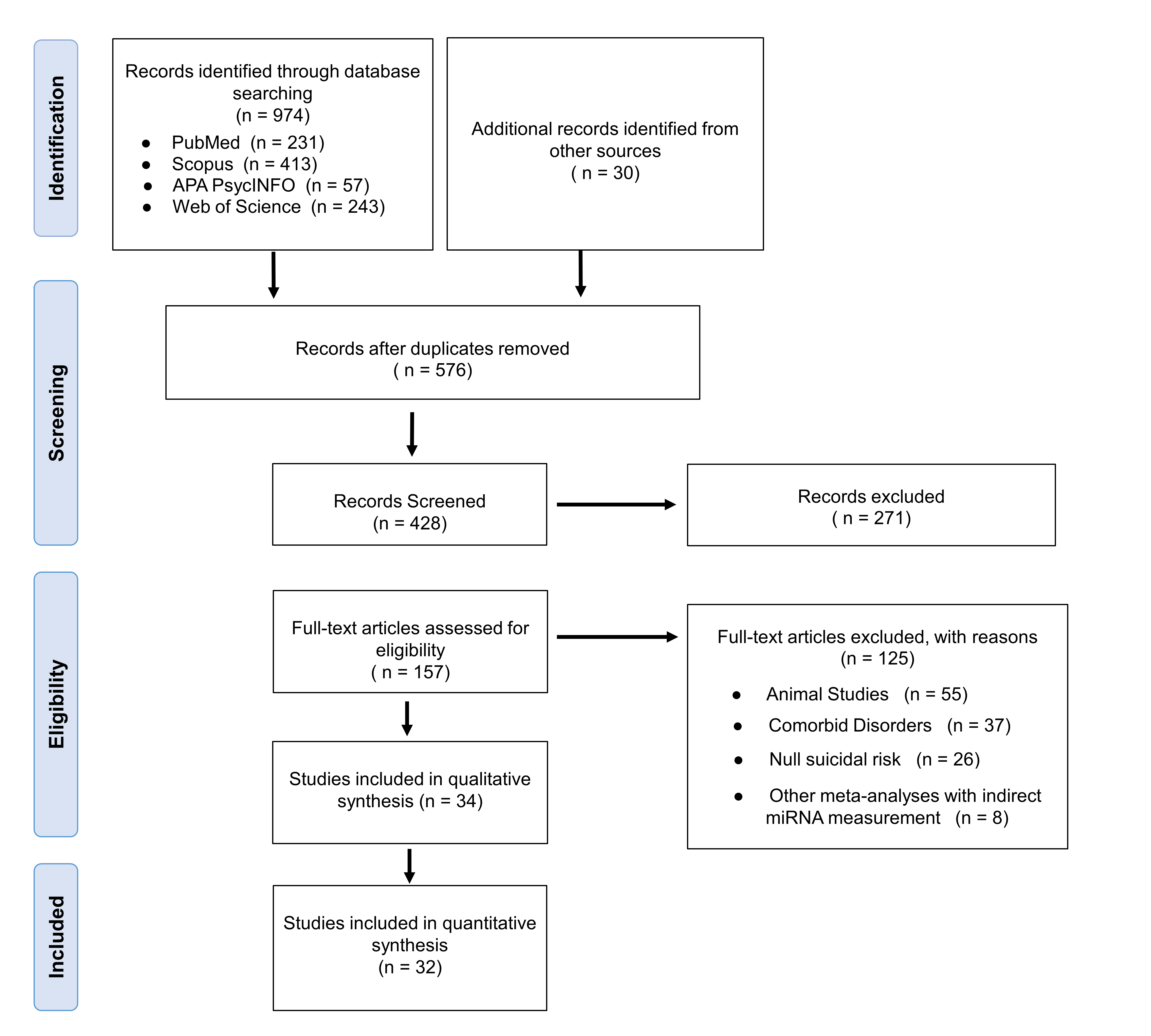

Supplement: Supplementary file 1 [file ijms-23-01294-s001.zip › Supplementary Figure S1.tif]
